# Dysregulated LINC01133 expression in laryngeal carcinoma: Prognostic implications and predicted ceRNA interactome

**DOI:** 10.22099/mbrc.2024.50390.1996

**Published:** 2025

**Authors:** Masoumeh Razipour, Zeinab Jamali, Saeed Sohrabpour, Farrokh Heidari, Maryam Lotfi, Elham Ghadami, Maryam Abtin, Mohaddese Maghsudlu, Leyla Sahebi, Abbas Shakoori

**Affiliations:** 1 Department of Medical Genetics, School of Medicine, Tehran University of Medical Sciences, Tehran, Iran; 2 Otorhinolaryngology Research Center, AmirAlam Hospital, Tehran University of Medical Sciences, Tehran, Iran; 3 Department of Pathology and Otorhinolaryngology Research Center, AmirAlam Hospital, Tehran University of Medical Sciences, Tehran, Iran; 4 Family Health Research Institute, Maternal-Fetal and Neonatal Research Center, Tehran University of Medical Sciences, Tehran, Iran; 5 Department of Medical Genetics, Cancer Institute of Iran, Imam Khomeini Hospital Complex, Tehran University of Medical Sciences, Tehran, Iran; # These two authors contributed equally to this work.

**Keywords:** Laryngeal Squamous cell carcinoma, Long non-coding RNA, LINC01133*LRRK2*, Competing endogenous RNA, Androgen Receptor

## Abstract

Long non-coding RNAs (lncRNAs) have recently emerged as critical regulators of oncogenic or tumor-suppressive pathways in human cancers. LINC01133 is a lncRNA that has exhibited dichotomous roles in various malignancies but to the best of our knowledge, the role of LINC01133 in laryngeal squamous cell carcinoma (LSCC) has not been previously investigated. This study aimed to investigate the expression, clinical significance, and potential functions of the LINC01133 in LSCC. Integrative bioinformatics analysis of sequencing data obtained from the Cancer Genome Atlas (TCGA) and Gene Expression Omnibus (GEO) datasets revealed LINC01133 as a differentially expressed lncRNA in head and neck/laryngeal cancers. Experimental validation via quantitative real-time PCR in 41 pairs of stage III and IV LSCC tissues and normal tissues adjacent to the tumor (NAT) demonstrated significant downregulation of LINC01133 in tumors (*p*<0.0001). Decreased LINC01133 expression associated with advanced tumor stage (*p*=0.0206) and lymph node metastasis (*p*=0.0203). The receiver operating characteristic analysis indicated potential diagnostic utility (AUC=0.7115, *p*=0.001). Bioinformatic predictions and literature mining suggested two potential competing endogenous RNA (ceRNA) mechanisms whereby LINC01133 may act as a tumor suppressor by sponging miR-205-5p to derepress the leucine-rich repeat kinase 2 (LRRK2) and androgen receptor, leading to dysregulation of cancer-related signaling cascades. This study provides initial evidence that loss of lncRNA LINC01133 expression may promote LSCC tumorigenesis, possibly by dysregulating microRNA interactions. Further verification of its regulatory mechanisms and diagnostic value is warranted.

## INTRODUCTION

Laryngeal squamous cell carcinoma (LSCC) represents the second most prevalent form of head and neck squamous cell carcinoma (HNSCC), accounting for approximately 95% of laryngeal malignancies. Despite advancements in surgery, chemotherapy, and radiotherapy, the clinical management of LSCC has not led to satisfactory patient outcomes, with over 180,000 new cases and nearly 100,000 deaths worldwide in 2020 [1, 2]. Therefore, it is crucial to understand the underlying mechanisms involved in the development and progression of LSCC, which can lead to identifying novel diagnostic, therapeutic, and prognostic biomarkers.

In recent years, long non-coding RNAs (lncRNAs) have emerged as one of the major regulators of cellular homeostasis and contributors to tumor biology across human cancers. LncRNAs are defined as non-protein coding transcripts greater than 200 nucleotides in length that regulate gene expression at various levels [3-6]. Numerous studies have revealed that lncRNAs play vital roles in cancer tumorigenicity and progression [7]. lncRNAs are aberrantly expressed in a variety of cancers and participate in many biological functions, such as cell proliferation, apoptosis, epithelial-to-mesenchymal transition (EMT), invasion, migration, angiogenesis, and differentiation [8, 9]. Despite various investigations on the function of lncRNAs in laryngeal cancer, the expression landscape and functional roles of many lncRNAs in the pathogenesis of LSCC remain largely unexplored. In the present study, focusing on the expression profiles of lncRNAs in The Cancer Genome Atlas (TCGA) repository and the Gene Expression Omnibus (GEO) database we performed an integrative data analysis to obtain differentially expressed (DE) lncRNAs, mRNAs, and related miRNAs. According to the results, LINC01133 lncRNA was selected for the next steps of the study.

Long intergenic non-coding RNA 01133 (LINC01133), located on chromosome 1q23.2, has gained considerable attention due to its dysregulated expression and involvement in numerous cancer types. LINC01133 has context-dependent activities in cancer, with evidence for both tumor- suppressive and oncogenic properties [10]. It is demonstrated that LINC01133 acts as a tumor suppressor in gastric cancer [11], nasopharyngeal cancer [12], oral [13] esophageal squamous cell carcinoma (ESCC) [14], and colorectal cancer [15]. However, studies have also shown potential oncogenic functions for LINC01133 whereby it promotes proliferation, invasion, and metastasis of hepatocellular carcinoma [16], renal cell carcinoma [17], cervical cancer [18], lung cancer [19], and pancreatic cancer [20] by modulating various pathways. The role of LINC01133 in ovarian [21, 22] and breast [23, 24] cancers remains contentious, as some studies suggest tumor-suppressive effects while others indicate possible pro-oncogenic activities. 

LINC01133 expression has been used as a biomarker to predict prognosis across various cancer types. However, as LINC01133 can have opposing roles in different tissues, the impact of its dysregulation on clinical outcomes is tissue-dependent [25]. For example, in gastric cancer [11], ESCC [14], and colorectal cancer [15], low LINC01133 levels correlate with lymph node metastasis (LNM) and advanced TNM stage. In contrast, increased expression of LINC01133 is associated with shorter overall survival and poorer prognosis in pancreatic [20, 26, 27] and lung cancers [19, 28]. The disparate relationships between LINC01133 levels and clinical outcomes highlight its complex, context-specific functions in human malignancies.

Exploring the molecular functions and clinical impact of LINC01133 may uncover new biomarkers and therapeutic approaches for laryngeal carcinoma. However, to the best of our knowledge, the expression of LINC01133 and its possible roles in LSCC remain unclear. In this study, we assessed LINC01133 expression in LSCC using quantitative real-time polymerase chain reaction (qRT-PCR) and examined its correlations with clinicopathological characteris-tics. Finally, using bioinformatics databases and systems biology approaches, we analyzed the potential roles of LINC01133 as a competing endogenous RNA (ceRNA). 

## MATERIALS AND METHODS

**Patients and tissue samples: **In this investigation, 41 pairs of LSCC tissue specimens at pathological stages III and IV, along with corresponding normal tissues adjacent to the tumor (NAT), were procured from the tumor bank of the Otorhinolaryngology Research Center at AmirAlam Hospital in Tehran, Iran. The study received approval from the ethical committee of the AmirAlam Hospital Complex (Ethical code: IR.TUMS.AMIRALAM.REC.1401.034). Additionally, written informed consent was obtained from all patients before their tissue samples being deposited in the tumor bank. Patients who had undergone any preoperative chemotherapy, radiotherapy, or immunotherapy prior to recruitment, as well as those with a history of previous or secondary malignancy, were excluded from the study. Samples were also evaluated by expert pathologists to determine the stages, based on clinicopathological characteristics.

**Transcriptome expression profiling in LSCC and HNSC through GEO and TCGA data analysis**

**Data source and analysis: **The TCGA-HNSC project transcriptomic data (https://portal. gdc.cancer.gov) was downloaded using the GDCRNATools package (R software). Data consisted of the Gene expression profile data (564 samples, 44 normal samples, and 520 primary tumors), miRNA expression profile data (567 samples, 43 normal and 524 primary tumors), and clinical data of patients. In addition, from the GEO database (https://www.ncbi. nlm.nih.gov/geo/), (GPL20301 platform), GSE130605 dataset which included sequencing data from the whole transcriptome of 50 LSCC and 50 paired NAT were analyzed using GEO2R. 

**Identification of DE mRNAs, DE lncRNAs and DEmiRs: **The TCGA-HNSC data was investigated to determine differentially expressed genes (mRNAs and lncRNAs) and miRNAs between tumor samples and normal head and neck tissue samples using R software, the limma package. Differentially expressed genes and miRNAs with log2 fold change |log2FC| ≥ 1 and adj.p.value <0.05 were considered for further analysis. The GSE130605 dataset was also analyzed to identify DE genes (mRNAs, lncRNAs, and miRNAs) between LSCC and paired NAT using GEO2R which utilizes the DESeq2. DE genes with the significance level cut-off < 0.05 and log2 fold change threshold ≥|1| were selected. To reduce false positive results, the results of the GSE130605 dataset analysis and TCGA-HNSC project were merged and intersection values were extracted using the Venn diagram tool (http://bioinformatics.psb.ugent. be/webtools/Venn/) and the final DEmRNAs, DElncRNAs, and DEmiRNAs were obtained. 

**Construction of ceRNA regulatory network hypothesis: **There were 16 DElncRNA obtained from previous steps and all of them upregulated in both HNSCC (TCGA-HNSC) and laryngeal data (GSE130605) except LINC01133. This lncRNA showed a contradictory expression in bioinformatics data and previously published studies. Therefore, LINC01133 was selected to construct the lncRNA/miRNA/mRNA network. 

**Expression Study** **and survival analysis: **Total RNA was extracted from 50 mg of fresh frozen tissue stored in liquid nitrogen using the Kiazol Reagent (KIAZIST Life Sciences, Iran) following the manufacturer’s protocol. The quantity and purity of the extracted total RNA were evaluated using a Nanodrop 2000C spectrophotometer (Thermo Scientific, USA). RNA integrity was assessed by visualizing the 28S:18S rRNA ratio via agarose gel electrophoresis. cDNA synthesizing from the isolated total RNA was performed using the ExcelRT^TM^ Reverse Transcription Kit II (SMOBIO Technology, Inc., Taiwan). The relative expression levels of lncRNA were assessed in both cancerous and non-cancerous tissues via qRT-PCR using the RealQ Plus 2x Master Mix Green (Amplicon, Denmark) on a Roche LightCycler® 96 System. The following cycling conditions were considered: initial denaturation at 95°C for 15 min, followed by 45 cycles of 95°C for 20 sec and 60°C for 50 sec. Primer specificity and reaction efficiency were evaluated via melt curve analysis (60-99°C) and agarose gel electrophoresis of the PCR products. The RPL30 gene was utilized as the reference for normalization of gene expression, as it has been identified as a suitable and stable housekeeping gene for quantitative gene expression analyses in HNSCC [29]. The primers used for LINC01133 and RPL30 were as follows: LINC01133-F: 5′-GGGGAGAGTAGGTGAAAAGATGA-3′, R: 5′-GCTGGACTTT GGAGAACTTTGC-3′; RPL30-F: 5′-TGGCTATCATTGATCCAGGTGAC-3′, R: 5′- GCAGG TTTAAGGTTTGCAGGTG-3′. The data for each sample was collected in duplicate. The 2^−ΔΔCT^ method was used to calculate the relative expression levels. Also, the correlation between LINC01133 expression and patient survival was determine using GEPIA2. 

**Constructing protein-protein interaction (PPI) network of the downregulated DEmRNAs: **The search tool for retrieval of interacting genes (STRING) (https://string-db.org) database was used to investigate the physical and functional interactions between downregula- ted mRNAs (proteins). The active interaction sources considered were according to text- mining, experiments, databases, coexpression, neighborhood, gene fusion, and cooccurrence and the minimum required interaction score was 0.400 for PPI network construction. The visualization of the obtained PPI network was done using Cytoscape software version 3.10. and the following criteria were considered for the selection of hub genes in the PPI network. 12 hub-genes genes with i) the highest degree (11-24) and ii) the highest corresponding BetweennessCentrality (0.04-0.1) were selected as downregulated hub-genes for further analysis. 

**Functional and pathway enrichment analysis of hub-genes: **Enrichr (http://amp.pharm. mssm.edu/Enrichr/) were used to investigate functional annotation and Kyoto Encyclopedia of Genes and Genomes (KEGG) pathway enrichment of 12 hubs downregulated common genes. P-value and adjusted p-value of <0.05 for KEGG pathway terms, were considered for selecting the significant terms respectively. 

**ceRNA network construction: **The potential interactions between LINC01133 and DEmiRNAs were predicted using RNA22 tool (https://cm.jefferson.edu/rna22/) and the interactions between DEmiRNAs and potential genes were also identified by miRtarbase (https://mirtarbase.cuhk.edu.cn/~miRTarBase/miRTarBase_2022/php/index.php). Subsequently, the DEmRNAs which are interacted with common DEmiRNAs in association with LINC01133 were selected. Cytoscape software (version 3.10.1). was used to construct the potential regulatory networks involved LINC01133/miRNA/mRNA axis. 

**Statistical Analysis:** In the current study, GraphPad Prism 9.1.0 software (GraphPad Software, Inc., San Diego, CA) was used for all statistical analyses. The data were expressed as mean ± standard deviation. Normality was assessed using the Kolmogorov-Smirnov test and Shapiro-Wilk test. The paired samples t-test was employed to compare the relative expression of lncRNA between LSCC tissues and matched NAT. The association between lncRNA expression level and clinicopathological characteristics was analyzed using the independent samples t-test. The receiver operating characteristic (ROC) curve was plotted to assess the diagnostic performance of studied lncRNA as a potential biomarker. P-values below 0.05 were considered statistically significant.

## RESULTS

**Figure 1:**
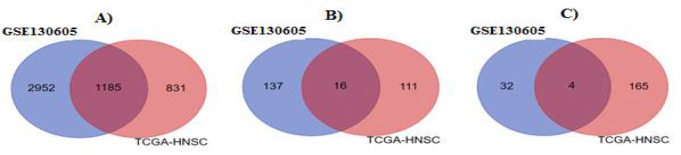
Venn diagrams of intersected deferentially expressed sequencing data. a. 1185 DE mRNAs (547 downregulated), b. 16 DElncRNAs (15 upregulated and one controversial LINC01133), and c. 4 DEmiRNAs. See also tables S1-S3.

The TCGA-HNSC project transcriptomic data and whole transcriptome sequencing of the GSE130605 dataset were analyzed. The transcriptomic data between tumoral tissues and normal or adjacent tissues revealed 4137 DE mRNAs, 153 DElncRNAs, and 37 DEmiRNAs in the GSE130605 dataset and 2016 DE mRNAs, 127 DElncRNAs and 169 DEmiRNAs in TCGA-HNSC project. After merging the sequencing data, the deferentially expressed results were as follows: 1185 DE mRNAs (547 downregulated), 16 DElncRNAs (15 upregulated and one controversial LINC01133), and 4 DEmiRNAs (3 upregulated and one downregulated) (Fig.1 and Tables S1-S3).

Considering the dysregulation pattern of DElncRNAs after analysis of two transcriptomic data, it is revealed that among 16 DElncRNAs only LINC01133 had a controversial expression pattern between two transcriptomic data. The results of several previous studies also indicated a double-edged sword-like properties for LINC01133 which can act as a tumor suppressor or an oncogene in a context-dependence manner. Fifteen other DElncRNAs demonstrated an upregulated expression pattern. So, in the current study, LINC01133/miRNA/mRNA regulatory network is considered for further analysis. 

**Figure 2:**
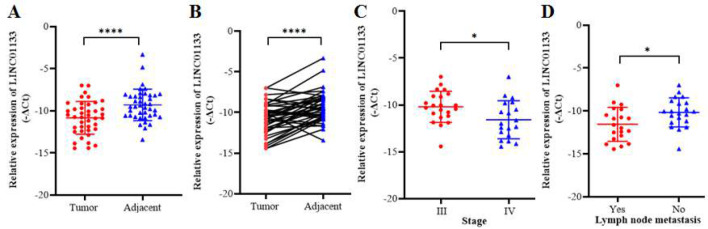
LINC01133 expression in LSCC. **A.** Lower expression of LINC01133 (****p<0.0001) in LSCC tissues compared with adjacent non-cancerous tissues. **B.** The corresponding relationship of LINC01133 expression between each pair of LSCC tissues and adjacent non-tumor tissues (****p<0.0001). **C.** LINC01133 expression was obviously lower in stage IV than in stage III (*p=0.020). **D.** Expression of LINC01133 was significantly lower in patients with lymph node metastasis (LNM) (*p=0.020).

LINC01133 expression levels were assessed in 41 LSCC tissues with stages III and IV and compared with their corresponding NAT. The results indicated a significant downregulation of LINC01133 expression in tumoral tissues compared to NAT (Mean of differences = -1.538 ± 1.896, *p*<0.0001) (Fig. 2A). Furthermore, the expression level of LINC01133 was remarkably lower in 73% (30/41) of tumor samples compared to that in NAT (Fig. 2B). The correlation between LINC0113 expression and patient survival in TCGA- HNSC was determine using GEPIA2 and is shown in Figure S2. 

The clinicopathological characteristics of the included patients are summarized in Table 1. These features include age, tumor size, tumor stage, histological grade, presence of LNM and lymphovascular/perineural invasion, and smoking/drinking status. As shown in Table 1, there is a significant association between decreased expression of LINC01133 and stage IV LSCC (*p*=0.0206) (Fig. 2C) as well as the presence of lymph node metastasis (*p*=0.0203) (Fig. 2D). No significant associations were observed between LINC01133 expression and other clinicopatho-logical factors, including age, tumor size, histological grade, and the presence of lymphovascu-lar and perineural invasion (*p*>0.05).

**Table 1:** Correlation of LINC01133 expression with clinicopathological features in LSCC patients

**Characteristics**	**No. of cases (%)**	**Difference between means (A-B) ± SEM**	**95% confidence interval**	***p*** **-value**
**Gender** Male Female	41 (100%) 0 (0%)	-	-	-
**Age (years)** < 57 ≥ 57	21 (51.22%) 20 (48.78%)	0.7708 ± 0.6037	-0.4502 to 1.992	0.209
**Tumor diameter (cm)** < 4 ≥ 4	21 (51.22%) 20 (48.78%)	-0.2869 ± 0.6144	-1.530 to 0.9559	0.643
**Differentiation** Well + Moderate Poor	28 (68.29%) 13 (31.71%)	-0.01158 ± 0.7441	-1.517 to 1.493	0.987
**Lymphovascular invasion** Yes No	13 (31.71%) 28 (68.29%)	0.09102 ± 0.6617	-1.247 to 1.429	0.891
**Perineural invasion** Yes No	17 (41.46%) 24 (58.54%)	-0.1156 ± 0.6249	-1.379 to 1.148	0.854
**Lymph node metastasis** Yes No	20 (48.78%) 21 (51.22%)	-1.390 ± 0.5745	-2.552 to -0.2276	0.020
**Clinical stage** III IV	22 (53.66%) 19 (46.34%)	1.390 ± 0.5761	0.2251 to 2.556	0.020
**Smoking** Yes No	37 (90.24%) 4 (9.76%)	-0.05865 ± 1.038	-2.158 to 2.041	0.955
**Alcohol consumption** Yes No	3 (7.32%) 38 (92.68%)	0.1057 ± 1.183	-2.286 to 2.498	0.929

**Figure 3:**
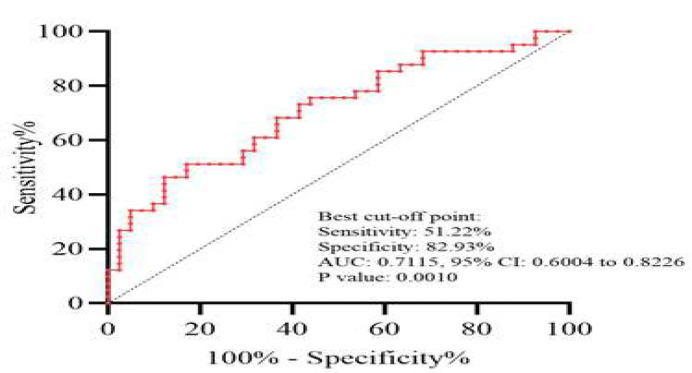
The receiver operating characteristic (ROC) curve of LINC01133 expression for discrimination of LSCC from adjacent tissues. AUC indicates area under the ROC curve.

To assess the potential diagnostic and prognostic value of the expression status of the studied lncRNA for distinguishing LSCC tissues from NAT, the ROC curve analysis was conducted (Fig. 3). The ROC curve analysis revealed that the LINC01133 expression level could be considered a promising discriminating marker for LSCC patients, with a sensitivity of 51.22% and specificity of 82.93% (AUC=0.7115, 95% CI=0.6004 to 0.8226, *p*= 0.0010). 

**Figure 4:**
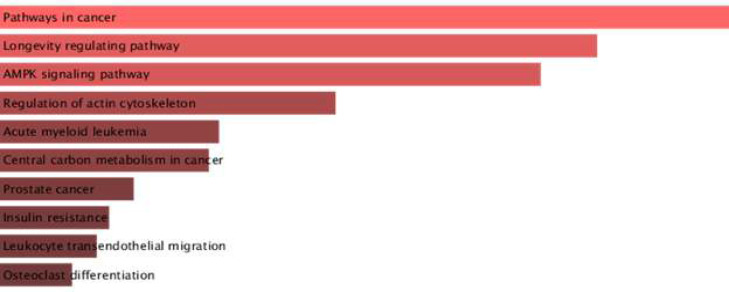
Enrichment analysis according to Enrichr database and according to the p-value for 12 hub-genes.

After the expression study, it was revealed that LINC01133 was downregulated in our samples. We hypothesize that there are some crucial downregulated hub genes that are regulated by some miRNAs and LINC01133 acts as a ceRNA related to these miRNAs. Accordingly, we considered the LINC01133/miRNA/mRNA regulatory network in which LINC01133 is downregulated and there are upregulated and downregulated miRNAs and mRNAs, respectively. The PPI network for downregulated common mRNAs was constructed using STRING. The resulting network included 546 nodes and 1192 edges (Fig. S1). In the next step, 12 Hub genes were identified according to the degree and Betweenness Centrality score using Cytoscape version 3.10.1 (Table 2). Enrichment analysis for functions and pathways of the hub genes revealed that they are active in pathways such as pathways in cancer, longevity regulating pathway, AMPK signaling pathway, prostate cancer, and regulation of actin cytoskeleton (Fig. 4).

**Table 2:** 12 Hub genes in the PPI network

	**Hub genes **	**Degree**	**BetweennessCentrality**
1	PPARG coactivator 1 alpha (PPARGC1A)	19	0.068002851
2	clusterin(CLU)	14	0.055599654
3	leucine rich repeat kinase 2(LRRK2)	11	0.046535115
4	elastin (ELN)	15	0.05588649
5	forkhead box A1(FOXA1)	13	0.054779315
6	androgen receptor (AR)	19	0.091613674
7	KIT proto-oncogene, receptor tyrosine kinase (KIT)	21	0.084867494
8	mucin 1, cell surface associated (MUC1)	21	0.062050042
9	myosin heavy chain 11(MYH11)	14	0.061951599
10	phosphoinositide-3-kinase regulatory subunit 1(PIK3R1)	18	0.079871036
11	peroxisome proliferator activated receptor gamma (PPARG)	24	0.107825272
12	C-X-C motif chemokine ligand 12(CXCL12)	24	0.076872587

**Figure 5:**
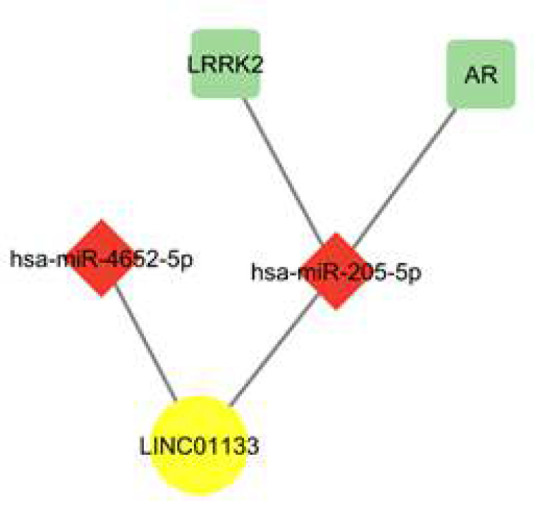
Two potential networks for LINC01133 activity

LINC01133- mediated ceRNA network was constructed based on the experimental results which approved the downregulation of LINC01133 in LSCC tissues in comparison to NAT. According to the RNA22 results, among 3 up-regulated common DEmiRNAs achieved in the bioinformatics phase, only the interaction of hsa-miR-205-5p, hsa-miR-205-3p, and hsa-miR-4652-5p with LINC01133 was approved. On the other hand, using miRtarbase it is revealed that among 12 hub-genes extracted from common downregulated DEmRNAs, AR and LRRK2 genes have interaction sites only with hsa-miR-205-5p. Accordingly, these two final networks can be proposed for LINC01133 activity in the context of LSCC development: LINC01133/hsa-miR-205-5p/LRRK2 and LINC01133 /hsa-miR-205-5p/AR (Fig. 5).

## Discussion

LSCC is one of the most prevalent forms of HNSCC [1]. LncRNAs are crucial regulators of gene expression in human malignancies through diverse mechanisms [4]. In particular, they can act as ceRNAs that sequester microRNAs and indirectly regulate target gene expression levels [30]. LncRNAs have been implicated as promising diagnostic and prognostic biomarkers across different types of cancers, including laryngeal cancer [4]. Many lncRNAs, such as FOXD2-AS1 [31], SNHG1 [32], and HOXA11-AS [33], have been shown to mediate therapy resistance, proliferation, and invasion in LSCC. Regarding LINC01133, contrasting effects in promoting or suppressing tumors across various cancer types have been reported [10, 34]. The impact of LINC01133 is believed to be influenced by its distinct interacting partners and signaling consequences in different tissues [10, 34]. Despite extensive research on dysregulated lncRNAs and their clinical utility as biomarkers, the expression and functional significance of LINC01133 in LSCC pathogenesis remain unexplored to the best of our knowledge.

In the current investigation, bioinformatics analysis revealed a contradictory expression pattern of LINC01133 between sequencing data obtained from TCGA-HNSC and GEO-LSCC datasets. LINC01133 expression was downregulated in the TCGA-HNSC dataset but upregulated in the GEO-LSCC dataset. This discrepancy might arise from potential biases in patient cohorts between TCGA-HNSC and GSE130605 datasets, including factors such as the tumor's origin, race/ethnicity, and other demographic or clinical variables. Additionally, technical issues, including platform variations, RNA contamination, or other experimental factors, could contribute to the observed inconsistencies. Furthermore, previous studies on other head and neck cancers, such as oral squamous cell carcinoma (OSCC) [13] and nasopharyngeal cancer [12], have reported downregulation of LINC01133 in tumor tissues compared to normal adjacent tissues, aligning with our bioinformatic finding from the TCGA-HNSC dataset. To elucidate the expression profile of LINC01133 and predict its possible role in laryngeal carcinoma, we performed expression analysis of LINC01133 in LSCC and adjacent tissues through qRT-PCR.

The expression analysis in 41 pairs of stage III-IV LSCC tissues and matched NAT showed significant downregulation of LINC01133 in tumor tissues versus NAT (*p*<0.0001). In the present study, significant associations were observed between low expression of LINC01133 and aggressive features of LSCC, including advanced TNM stage (*p*=0.0206) and LNM (*p*=0.0203), indicating its tumor suppressive function in LSCC. This aligns with some prior studies in other malignancies where LINC01133 inhibited proliferation, invasion, and treatment resistance [11-15]. The ROC curve analysis indicated that LINC01133 can be considered as a promising discriminatory biomarker in LSCC. If these findings are validated in clinical settings, assessing the expression level of this gene could assist in prognosis prediction and guide therapeutic decision-making.

In the subsequent step, considering the downregulation of LINC001133 in LSCC tissues at the experimental level, further bioinformatics analysis was performed to investigate the hypothetical molecular mechanism of LINC01133’s function as a ceRNA. Two hypothetical pathways were proposed, including the LINC01133/hsa-miR-205-5p/LRRK2 and the LINC01133 /hsa-miR-205-5p/AR pathways. 

MiR-205-5p displays heterogeneous dysregulation across various cancer subtypes, acting either as an oncogene or tumor suppressor through various downstream mechanisms [35]. This miRNA, known as a marker of epithelial phenotype, has been observed to decrease during the process of EMT, a crucial stage in facilitating tumor invasion and metastasis. Mechanistically, it can target EMT transcriptional regulators like ZEB1/ZEB2 to inhibit EMT [35, 36]. However, in endometrial carcinoma [37, 38], ovarian cancer [39, 40], and nasopharyngeal carcinoma [41, 42], miR-205-5p is overexpressed and promotes malignant phenotypes. In endometrial cancer, it targets tumor suppressors PTEN and ESRRG to inhibit apoptosis and drive proliferation [37, 38]. In nasopharyngeal carcinoma, the overexpression of miR-205 leads to PTEN down-regulation and the activation of the AKT pathway. This process enhances tumor progression and contributes to resistance against radiation therapy [41, 42]. Interestingly, in the lung [43] and esophageal [44] cancers, the high miR-205-5p expression indicates squamous cell carcinoma whereas low expression marks adenocarcinoma, highlighting utility as a diagnostic marker. There is contradictory evidence for miR-205 expression in LSCC. Tian et al. demonstrated that miR-205 could suppress proliferation and promote apoptosis in LSCC [45]. Conversely, other studies have reported elevated levels of miR-205 in HNSCC [46] and in LSCC, where it promotes cell proliferation and invasion by downregulating CDK2AP1 expression [47] or through AKT-mediated EMT [48].

Consistent with our bioinformatic findings, some other investigations have revealed that miR-205 can be a negative regulator for the expression of Leucine-rich repeat kinase 2 (LRRK2) [49-51]. LRRK2 is a large, multi-domain protein that exhibits both GTPase and kinase enzymatic activities. As a result, this protein engages in multifunctional activities through interactions with various proteins and the display of catalytic functions as a GTPase and kinase. LRRK2 is found to act in several key cellular activities, primary stages of autophagy, endocytosis, and the functions related to mitochondria and cytoskeleton [52, 53]. Substantial studies revealed the association of LRRK2 genetic variations with Parkinson’s disease as well as immune-mediated conditions like inflammatory bowel disease, leprosy, and tuberculosis [52, 53]. In recent years, several studies have identified LRRK2 mutations or dysregulation of its expression in various types of human malignancies including thyroid cancer [54], intrahepatic cholangiocarcinoma [55], lung cancer [56, 57], and renal carcinoma [58]. Intriguingly, in a cancer-type dependent manner, LRRK2 appears to affect tumor development -both promoting and suppressing them- by affecting crucial cellular pathways [53]. Proposed mechanisms for a potential LRRK2 tumor suppressor function include the phosphorylation of p53 [59] and activation of ERK and JNK-dependent autophagy [60]. In contrast, LRRK2 may also have an oncogenic activity via MET signaling induction [58]. Notably, in ovarian cancer, elevated miR-205 levels are associated with a decrease in its lncRNA sponge LINC01133. This imbalance leads to reduced expression of the miR-205 target LRRK2 and enhances the proliferative, migration, and invasion of ovarian cancer cells. Thus, the LINC01133/miR-205/LRRK2 axis represents a novel pathway driving ovarian cancer pathogenesis [21]. According to our bioinformatic analysis, dysregulation of this pathway could also contribute to LSCC oncogenesis. However, further validation analyses examining the expression and functional impacts of the LINC01133/miR-205/LRRK2 network specifically in LSCC models are needed. Elucidating whether this signaling cascade is truly aberrant and a driver in LSCC pathogenesis could unveil new prognostic or therapeutic opportunities.

As previously described, our bioinformatic analysis predicted another potential ceRNA network involving LINC01133, hsa-miR-205-5p, and the androgen receptor (AR). In this hypothesized pathway, LINC01133 acts as a ceRNA that sequesters hsa-miR-205-5p, effectively derepressing AR, a validated target of hsa-miR-205-5p. Several studies have demonstrated that miR-205 directly targets and downregulates AR expression across different cell types [61, 62]. AR is a sex steroid hormone receptor that is activated by ligands such as dihydrotestosterone (DHT) and plays a key role in multiple physiological processes, such as reproductive system development. In its inactive state, AR is stabilized in the cytoplasm through binding to chaperone proteins. Upon ligand binding, AR dissociates from chaperones, after conformational changes, it moves to the nucleus and binds DNA at androgen responsive elements (AREs) to modulate target gene expression. In addition to this genomic signaling, AR can activate non-genomic cascades like the PI3K/AKT pathway through cytoplasmic interactions [63]. Given its influence over various oncogenic mechanisms including the PI3K/AKT, EGFR, Src, and WNT pathways, AR dysregulation plays crucial roles in promoting the proliferation and progression of certain cancers such as prostate, breast, liver, and ovarian malignancies [63, 64]. While the oncogenic functions of AR signaling have been well documented, numerous evidence indicates AR can also exhibit tumor-suppressive roles in certain cancer contexts. For example, studies in prostate cancer [65-70], renal cell carcinoma [71], liver cancer [72], and breast cancer [73-75] have shown that AR activation can suppress cell proliferation, migration, and metastasis or induce apoptosis and differentiation programs counteracting malignant phenotypes. The exact mechanisms determining AR's dichotomous context-dependent roles are not fully understood but may relate to differences in interacting cofactors, relative expression of AR variants, epigenetic and post-translational control of AR activity, crosstalk with other cellular signaling networks, and influence of the tumor microenvironment. Further investigation is warranted to elucidate the specific factors that dictate when AR signaling promotes or impedes tumorigenesis across distinct cancer types.

AR signaling has been implicated as potentially playing a role in HNSCC; however, its mechanism of action remains poorly understood [76, 77]. While some studies have reported upregulation of the AR in HNSCC [78, 79], other analyses [80, 81], as well as our bioinformatic findings, indicate that downregulation of AR can also occur in HNSCC compared to normal tissue. Additionally, it has been reported that AR expression is significantly lower in poorly differentiated laryngeal SCC and cases with lymphatic invasion, indicating low AR may promote more aggressive carcinoma progression [82]. Overall, the available evidence highlights the complex, context-specific nature of AR signaling in HNSCC/LSCC pathogenesis.

In summary, the lncRNA LINC01133 appears to act as a tumor suppressor in LSCC. Our experimental analysis revealed that LINC01133 is downregulated in LSCC tissues compared to NAT. Decreased expression associated with the presence of LNM and advanced TNM stages supporting its inhibitory impact on aggressive cancer phenotypes. Bioinformatics prediction and literature evidence suggest the tumor suppressive functions of LINC01133 may occur through sponging miR-205-5p to derepress the miR-205-5p targets LRRK2 and AR. Additional studies validating and exploring these ceRNA networks would offer further insight into LSCC pathogenesis. Given its dysregulation in LSCC and correlation with unfavorable prognostic features, LINC01133 merits investigation as a novel biomarker or even a potential therapeutic target in laryngeal carcinoma. Overall, this study unveils the clinical relevance and hypothetical molecular mechanisms of LINC01133 in LSCC that warrant future research. 
