# Supplementary material for: Dysregulated LINC01133 expression in laryngeal carcinoma: Prognostic implications and predicted ceRNA interactome
Source: Mol Biol Res Commun. 2025;14(1):93–107. doi: 10.22099/mbrc.2024.50390.1996 (PMC11624609; doi:10.22099/mbrc.2024.50390.1996)
Supplement: Supplementary file 1 — Figure S1 [file mbrc-14-93-s001.pdf]

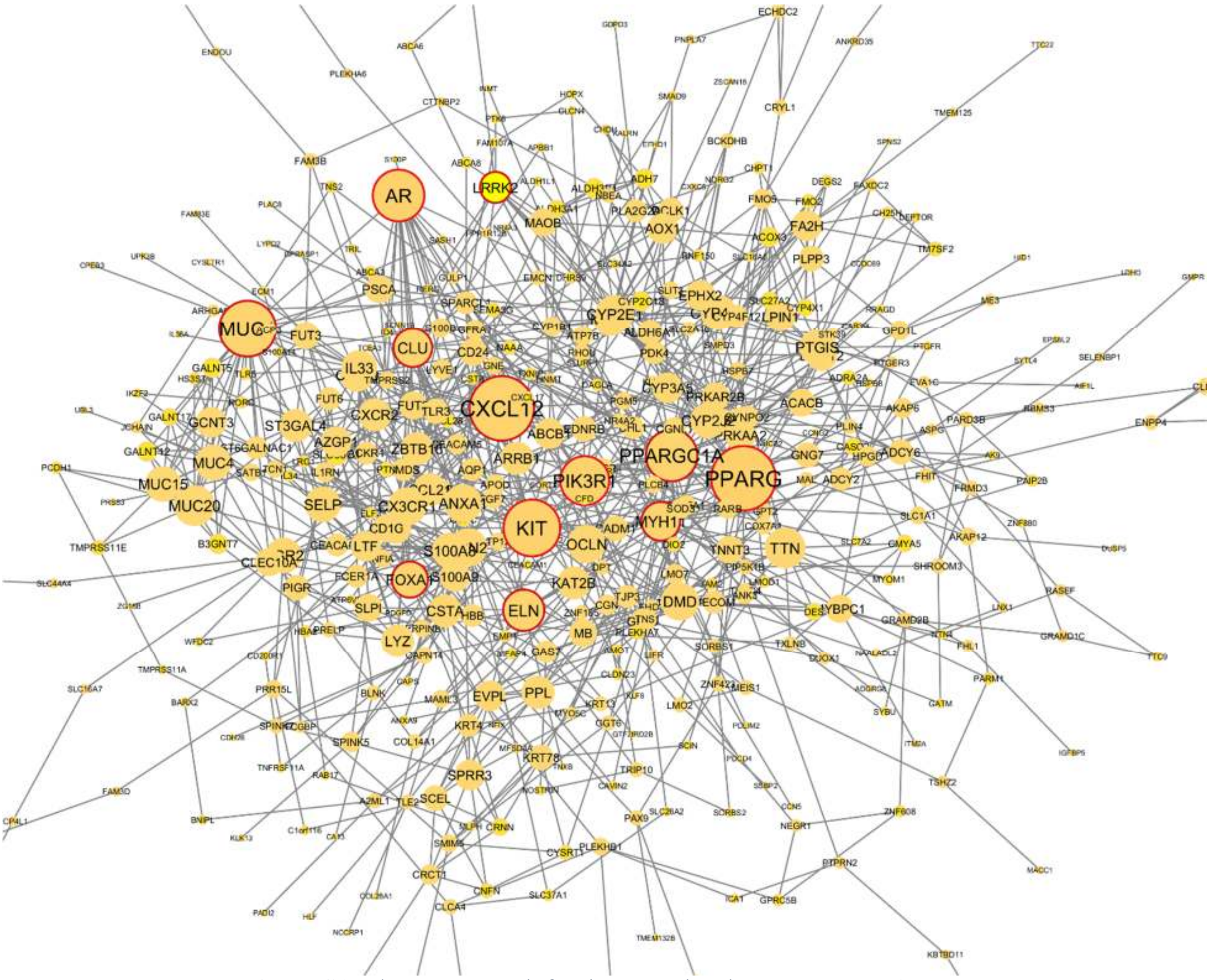

Figure S1: The PPI network for downregulated common mRNAs

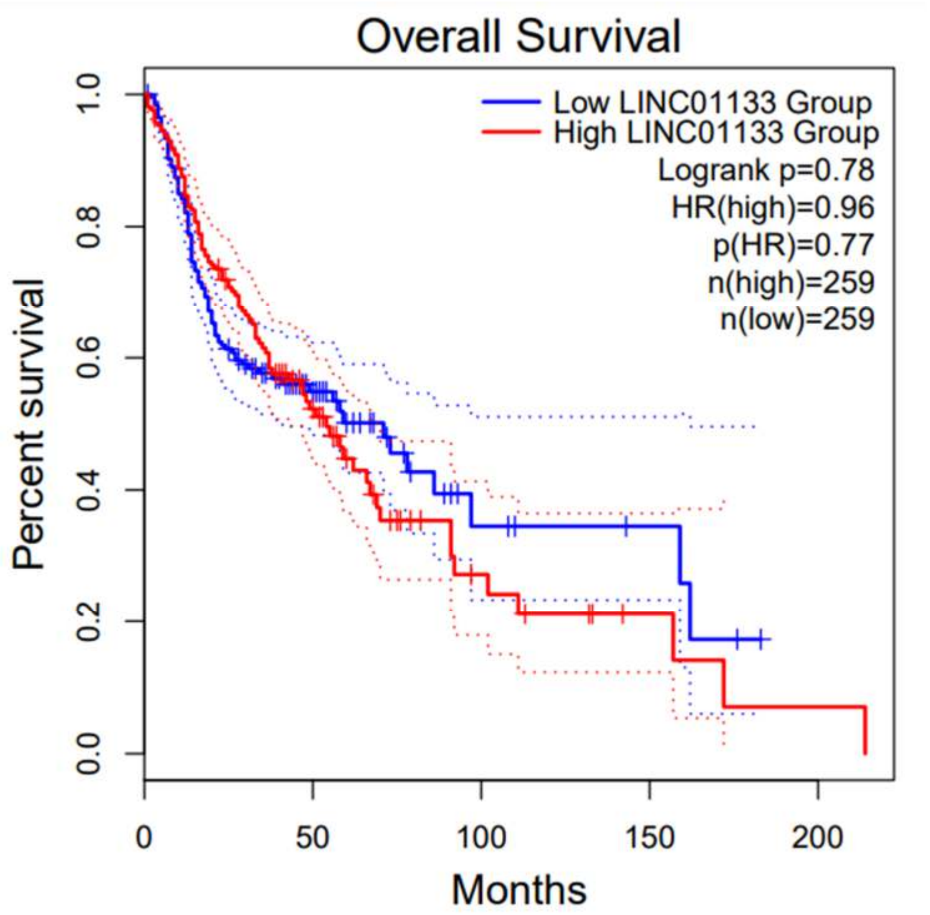

Figure S2: The correlation between LINC0113 expression and patient survival.
